# Metaphors of adolescence during COVID-19 pandemic: a mixed-method analysis in relation to well-being and alexithymia

**DOI:** 10.3389/fpsyg.2024.1355752

**Published:** 2024-03-14

**Authors:** Eleonora Farina, Alessandro Pepe

**Affiliations:** Department of Human Sciences for Education “Riccardo Massa”, University of Milano Bicocca, Milan, Italy

**Keywords:** well-being, alexithymia, self-representation metaphors, adolescents, mixed-method

## Abstract

**Introduction:**

During the pandemic, young people experienced a general increase in stress levels in their home and school environments and in their relationships with peers and family, largely due to restrictions on freedom of movement and social isolation. The ability to identify sources of stress and respond positively to them, using both personal and environmental resources, seems to be key to maintaining an acceptable level of well-being. This study investigates the association between alexithymic traits, self-perceived well-being, and self-representations in adolescents as expressed via narrative metaphors during the COVID-19 epidemic.

**Methods:**

The sample comprised 229 Italian adolescents (51.1% females, mean age = 16.64). The research design was based on an exploratory, parallel, mixed-method approach. A semi-structured online interview was used as the major data gathering tool including both standardized quantitative questionnaire and open-ended questions. Data were analyzed by means of descriptive statistics, quantitative textual analysis and multidimensional co-word correspondence analysis.

**Results:**

Main findings reveal a general low level of perceived well-being associated with alexithymia, affecting adolescents’ lexical choices for their metaphors. Alexithymia-related low levels of well-being correspond to metaphors in which confusion and overpowering emotions predominate. Vivid pictures indicating vitality and a bright view on the future is often correlated with high levels of well-being.

**Discussion:**

Overall, these novel findings appear to show an interactive effect of perceived well-being and alexithymia on adolescents’ ability to identify and describe their own condition. Furthermore, metaphors emerge as powerful tools for investigating well-being in adolescents since closely related to inner states.

## Introduction

The COVID-19 pandemic forced the implementation of multiple measures aimed at limiting the spread of the disease. These included closing places of public recreation and education, such as schools and universities, and restricting face-to-face interaction by means of enforced “social distancing.” Adhering to social distancing has been particularly challenging for adolescents, for whom interaction with peers is especially important. Adolescence represents a period of rapid physical, social, cognitive, and emotional change with key implications for health and well-being into adulthood (Azzopardi et al., 2019). Some of the most notable changes relate to an increased focus on developing friendships and interacting with peers (Spear, 2000; Casey et al., 2010; Blakemore and Mills, 2014) and a reduction in time spent with parents, also due to the development of greater autonomy and the changing nature of child–parent relationships (Larson et al., 1996; Laursen and Hartl, 2013).

Although adolescence can be a time filled with new and exciting experiences, it can also be a period of great difficulty and manifold social challenges. Experiences undergone during this period can greatly influence and shape an individual’s future social interactions and overall psychosocial well-being. Established evidence indicates that the incidence of most mental health difficulties peaks during the transition from childhood to young adulthood, affecting up to 20% of adolescents (Bertha and Balázs, 2013). Furthermore, among under-25 s, psychological and emotional difficulties account for almost one in two instances of health loss (Kessler et al., 2007; Gore et al., 2011). A recent survey on health and well-being in European adolescents (Inchley et al., 2020) yielded key findings: there is a general decline in mental well-being with increasing age, whereby older adolescents have lower levels of life satisfaction, are less likely to report excellent health, and suffer more frequent health problems. By age 15, girls report poorer mental well-being than boys. The prevalence of multiple health complaints has increased since 2014, with the most common concerning nervousness, irritability, and sleep difficulties. The key protection factors that emerged from the study were parents and good communication with parents and the peer group. A recent study by Campbell and Osborn (2021) drew on network analysis to examine the relationship between psychopathology (in terms of depression and anxiety symptoms) and psychological well-being (in terms of happiness, optimism, social support, perceived control, and gratitude) in adolescence. Emotional help and support from family and loved ones during adolescence seemed to be the key to coping with negative psychopathological symptoms, while low interest/pleasure emerged as a symptom influencing the relationship between well-being and psychopathology: the authors postulate that having low interest/pleasure in everyday things may lead adolescents to live a withdrawn life lacking the social support needed to improve positive well-being.

Pre-pandemic research has therefore shown that family and school connections are leading protective factors for the mental health of adolescents and young people. During the pandemic, young people experienced a general increase in stress levels in their home and school environments and in their relationships with peers and family, largely due to restrictions on freedom of movement and social isolation. In spring 2020, high school students did not have access to school premises and were invited to participate in various forms of distance learning. In many cases, the school closures continued into autumn/winter 2020 and spring/summer 2021, with adolescents being afforded variable forms of access to teaching (fully virtual, partially in-person, or – in a smaller proportion of cases – fully in-person). At the same time, adolescents’ access to social support networks such as peers or relatives was significantly reduced. These changes in teenagers’ living environments affected their mental health and well-being, with a general increase of post-traumatic stress, anxiety, depressive symptoms, insomnia, denial, anger, and fear, as well as grief-related symptoms (Duan et al., 2020; Guessoum et al., 2020) and a worsening of existing mental health problems (Golberstein et al., 2020). A number of studies have attempted to identify individual differences among adolescents in terms of how successfully they managed their well-being during the pandemic, for example, in relation to the tendency to use certain coping strategies. A recent study with Italian adolescents (Pigaiani et al., 2020) showed that one in two adolescents experienced a significant deterioration in their perceived levels of well-being during the pandemic, with an increase in anxiety symptoms. The most interesting finding concerned the difference between adolescents who reported having more frequently adopted active coping strategies and identified positive aspects of the situation and those who displayed poorer self-regulation skills and suffered more acutely from isolation and living full-time with their families: the former group did not detect as great a decrease in their levels of well-being as did the latter. The ability to identify sources of stress and respond positively to them, using the available personal and environmental resources, seems therefore to be key to maintaining an acceptable level of well-being even in the face of stressful events.

### Alexithymia in facing stressful events

Given that past research strongly suggests that adolescents’ interactions with their peers, friends, and family can have a major impact on their psychological well-being – especially in the face of stressful events such as the recent pandemic – it is important to investigate the factors that may contribute to relationship difficulties during this vulnerable stage of life. Among such factors, the ability to effectively manage emotions is fundamental to healthy psychological and social development in adolescence. Furthermore, the issue of emotional regulation is particularly salient during this life stage, which is characterized by a generalized and temporary increase in the use of maladaptive strategies, linked partly to neuroendocrine maturation processes and partly to the transition from hetero-regulation (implemented by caregivers) to emotional self-regulation strategies (Zimmermann and Iwanski, 2014). With regard to the use of maladaptive emotion regulation strategies, alexithymia has been found to be a strong predictor: this personality construct (Sifneos, 1973) consists of difficulty in identifying and verbalizing emotions, impoverishment of the imagination, and poor symbolic thinking skills. These deficits are believed to cause an inability to regulate emotions and affect, predisposing individuals to psychological and somatic symptoms.

In the literature, alexithymia has been associated with lower perceived social support (Kojima et al., 2003; Karukivi et al., 2011), a range of mental health problems, including depression, anxiety and eating disorder symptoms (Karukivi et al., 2010a,b), psychological distress (Saikkonen et al., 2018), and poor quality of life (Mattila et al., 2009). Not surprisingly therefore, alexithymia is associated with difficulty in coping with stressful events and the chronicization of dysfunctional responses (Mavroveli et al., 2007; Fiorilli et al., 2020). Indeed, in line with the stress-alexithymia hypothesis proposed by Martin and Pihl (1985), alexithymic traits can negatively affect the ability to identify and cope with stressors. In turn, this difficulty can prolong exposure to stressful situations and favor the emergence of burnout symptoms (Lapa et al., 2017; Romano et al., 2019), including in high school students (Fiorilli et al., 2020; Farina et al., 2021). Adolescents with alexithymic traits may be more prone to incorrectly assessing or failing to identify emotionally stressful elements of problematic situations, which in turn may heighten and protract their perceptions of tension and difficulty, ultimately leading them to become emotionally exhausted (Hamaideh, 2018). Furthermore, teenagers who find it difficult to identify and manage their emotions generally express more anger, an emotion that is associated with engaging in aggressive behavior, whether physical or verbal (Sfeir et al., 2020; Pepe et al., 2023). Therefore, if we take the coronavirus emergency to represent a source of multiple, intense stressors, it is plausible that presenting with alexithymic traits may be a strong risk factor for adolescents’ well-being during this period. This is in line with recent findings reported in the literature: studies found that alexithymic traits predicted psychopathological symptoms triggered by the COVID-19 scenario (Tang et al., 2020; Merlo et al., 2021). Alexithymia was also found to significantly mediate the effects of attachment to mothers and attachment to peers on peritraumatic distress due to COVID-19 (Tambelli et al., 2021). Neurobiological studies had previously identified an association between alexithymia and the dysregulation of cortisol levels in response to stressful situations (Alkan Härtwig et al., 2013), which drives higher anxiety and depressive symptoms. Wang et al. (2021) reported a significantly higher incidence of alexithymia in adolescents suffering from depression during the pandemic. These outcomes suggest that the public health emergency has been a major source of stress, which has facilitated the development of alexithymia by negatively influencing the identification, expression, and regulation of emotions during everyday life events. In turn, personality traits or behaviors associated with alexithymia affect the physical and mental health of adolescents – especially the most fragile – reducing their capacity for social adjustment and increasing their risk of psychopathological outcomes.

Thus, it can be assumed that alexithymia is associated with poor capacities of identifying and describing both internal states and stressful external events and is plausibly one of the major risk factors for well-being in adolescence. Furthermore, subjective well-being in adolescence may be associated with better self-assessment and understanding of one’s own state of health (Katja et al., 2002). It is therefore important to explore the relationships between alexithymia and adolescents’ perceptions of themselves and their levels of well-being during the COVID-19 emergency. Metaphors have emerged as valuable tools in understanding the complex cognitive and emotional landscape of adolescents. Lakoff and Johnson (1980) argue that metaphors are not merely linguistic embellishments but fundamental mechanisms through which individuals conceptualize abstract concepts. The application of self-metaphors in adolescent research aligns with the cognitive development theories, emphasizing the role of metaphorical thinking in shaping cognitive processes during adolescence and is also widely used in narrative counseling (Larsen and Larsen, 2004). Therefore, the integration of self-metaphors in research methodologies offers a nuanced approach to capturing the intricate nuances of adolescent experiences, enriching our understanding of their cognitive and emotional development.

In this study, we set out to investigate adolescents’ perception of themselves and their levels of well-being during the COVID-19 pandemic. More specifically, we firstly studied the association between alexithymic traits and self-perceived well-being. According to abovementioned literature, we hypothesized to find a negative association between these variables. Furthermore, we explored possible relations between well-being, alexithymia, and self-perceptions during adolescence as expressed by means of narrative metaphors. Besides assessing participants’ levels of well-being by means of a validated and standardized instrument such as the WHO-5, we assumed that teenagers’ use of words to describe themselves would be associated with and – at the same time – act as a good indicator of their ability to identify and describe their inner states as well as external stressful events, and ultimately reflect their state of well-being. We hypothesized that low alexithymia would be associated with high perceived well-being and positive, pro-active narrative metaphors; on the contrary, we expected that high alexithymia and low well-being would be more strongly associated with “passive” and negative images of adolescence (for example, stressful difficulties and obstacles).

## Method

### Sample

The sample comprised 229 Italian adolescents attending classic and scientific high schools (e.g., in the Italian educational system this kind of school is called *lyceum*). The schools were located in a highly urbanized area, near the city center. The gender composition of the sample was evenly balanced, with 48.9% males (*n* = 112) and 48% females (*n* = 110); 7 participants (3.1%) did not state their gender. Age ranged from 14 to 19 years (*M* = 16.64, *SD* = 1.46). We adopted a convenience sampling approach (Emerson, 2015). Criteria for being included were: (1) attending high school, (2) being aged between 14 and 19 years, (3) accepting the terms of participation in the study. We set no exclusion criteria. We collected the data over a two-month period, from April to June 2021.

### Procedure and materials

The research design was based on an exploratory, parallel, mixed-method approach (Teddlie and Tashakkori, 2006; Anguera et al., 2020). The primary data collection instrument was a student-centered, computer-assisted, and semi-structured web interview (CAWI; Kurniawan, 2018). The interview consisted of three main sections: (1) demographic background, (2) quantitative measures of wellbeing and alexithymia, (3) open-ended question on being an adolescent during the COVID-19 public health emergency. Each section included both closed questions (e.g., statements to be rated on Likert scales) and open-ended questions, with a view to triangulating the emergent data and cross-validating the results (Denzin, 2012). The data were collected anonymously, and all participants were fully briefed about the nature of the research. Participation in the study was on a voluntary basis, meaning that no monetary or financial rewards were offered to the participants. The study was approved by the Ethical Board of Milano-Bicocca University (prot. N. 0059806/21) and it was conducted according to the ethical principles defined by the Declaration of Helsinki (World Medical Association, 2001) and the American Psychological Association code of conduct (American Psychological Association, 2010). Given that the sample was composed of minors, we also obtained informed parental consent for all participants.

### Measures

#### World Health Organization Well-Being Index

The five-item World Health Organization Well-Being Index (WHO-5, Staehr, 1998) is a brief, generic global rating scale measuring subjective well-being (Topp et al., 2015). The measure has been used in many different settings as a measure of positive well-being and a proxy for mental health (Jahoda, 1958). The questionnaire contains five items: (1) ‘I have felt cheerful and in good spirits’, (2) ‘I have felt calm and relaxed’, (3) ‘I have felt active and vigorous’, (4) ‘I woke up feeling fresh and rested’ and (5) ‘My daily life has been filled with things that interest me’. Participants must rate these statements on a Likert-type response scale ranging from 5 (all of the time) to 0 (none of the time). We computed raw WHO-5 scores by summing the scores for responses to individual items. Global scores ranged from 0 (absence of well-being) to 25 (maximum well-being) and we then conventionally translated them to a scale from 0 to 100. A generally accepted threshold for identifying reduced wellbeing and being at risk of developing symptoms of depression is a score of under 50 (Hoffman et al., 2012). In the present study, the reliability coefficient for the WHO-5 measure – as assessed via Cronbach’s alpha (α; Cronbach, 1951) – was 0.817.

#### Toronto Alexithymia Scale

The Toronto Alexithymia Scale (TAS-20, Bagby et al., 1994; Italian validation by Bressi et al., 1996) consists of 20 items assessing three dimensions: difficulty identifying feelings (DIF; sample item: “I am often confused about what emotion I am feeling”), difficulty describing feelings (DDF; sample item: “It is difficult for me to reveal my innermost feelings even to close friends”), and externally-oriented thinking (EOT; sample item: “I prefer to analyze problems rather than just describe them”). Students are asked to express their level of agreement with each item by rating it on a 5-point Likert scale (from 1 = strongly disagree, to 5 = strongly agree). Both the original validation study (Bagby et al., 1994) and the validation study of the Italian version (Bressi et al., 1996) confirmed the three-dimensional structure of the TAS-20 but recorded better reliability for the global measure. For this reason, in the present study – as in numerous others in the literature (e.g., La Ferlita et al., 2007) – TAS-20 was used as a global unidimensional measure of alexithymia (Cronbach’s alpha = 0.852). Additionally, the adoption of a global score allowed us to use standard cut-off points for classification purposes. Specifically, following Bagby et al. (1994), we took a score of ≤50 to indicate “non-alexithymia,” between 51 and 60 to indicate “borderline alexithymia,” and over 61 to indicate “alexithymia.”

#### Metaphors as qualitative research material

Language functions as both a subject and a medium in virtually all qualitative and mixed-method research approaches (Schmitt, 2005). Within such approaches, the use of metaphor is frequently seen as either a literary device or a distinctive characteristic of figurative language (Lakoff, 1986) given that metaphors can reveal and represent a participant’s ideas and perceptions, as well as fulfilling a generative or catalytic function. For instance, during the data collection phase of a study by Henri and Hay (1997), metaphors were elicited as proxies for mental schema. The authors invited respondents to complete the phrase “a teacher-librarian is like...” and then describe what they had just written. Analyzing this data enabled Henri and Hay to identify the underlying images and understandings that their respondents were brining to bear on their professions, which in turn shed light on structural and cultural impediments to librarians becoming teacher-librarians. Thus, metaphors may offer a valuable tool for depicting complex realities (Miles and Huberman, 1994), bring to light previously unnoticed aspects of phenomena (Lakoff and Johnson, 1980), and add depth of meaning to existing knowledge. In addition, the use of metaphors can help to apply established properties of familiar notions to less familiar occurrences, so that less familiar phenomena can be investigated and elucidated (Moss et al., 2003). Finally, by reducing concepts and highlighting some features over others, metaphors can bring behaviors and processes into sharper focus (Lakoff and Johnson, 1980). In the present study, we surveyed adolescents’ metaphors by asking the participants “If you had to describe using one phrase, image, or metaphor what it is like to be a girl/boy your age these days, what would you write?”

### Data analysis strategy

We subjected the data from this mixed-method study to quantitative textual analysis (QTA; Bolden and Moscarola, 2000; Kelle, 2004). QTA is a branch of qualitative content analysis and assumes that (1) words that tend to appear together (i.e., in close proximity) in a given context may be interpreted as related to the same lexical theme or concept within the discourse under study (Brier and Hopp, 2011) and (2) traditional statistical techniques may be used to analyze narrative data (Miller and Riechert, 2001). We applied co-word analysis (CA, Greenacre and Blasius, 2006) to the adolescents’ responses to the question “If you had to describe, using one phrase, image, or metaphor, what it is like to be a girl/boy your age these days, what would you write?” The value of using CA to analyze this type of material is that it enables researchers to represent the structure of their data by converting a set of proximity measures into visual distances corresponding to specific locations within a spatial (Cartesian coordinate system) configuration (Borg et al., 2013). The outputs of this analysis are word-based maps that help to identify recurrent themes, their relative importance, and how they are related to one another. In this study, we assessed lexical similarities within our narrative corpus in terms of the relative chi-square and Salton’s cosine values (Hamers, 1989) along with their statistical significance levels (cut-off was set at *p* < 0.05). Salton’s cosine allowed us to organize these relationships geometrically so that “they can be visualized as structural patterns of relations” (Wagner and Leydesdorff, 2005, p. 208).

In the context of the present study, we expected that the output of the CA would allow us to analyze the adolescents’ metaphors by grouping together “naturally” occurring themes based on lexical similarity measures; we would then be able to examine how the prevalence of these themes was related to the participants’ levels of well-being and alexithymia. All analyses were conducted using TLAB 5.0 and SPSS 21.0.

### Data cleaning and general descriptive statistics for the text corpus

As with other data exploration techniques, QTA requires a pre-processing stage to prepare the data for analysis. In keeping with the literature, we applied *normalization* (removing all general function words such as articles, connection forms, and prepositions); *lemmatization* (reducing all inflected words to their root form as it would be found in the dictionary) and *synonimization* (words that may be considered equivalent from the semantic point of view– e.g., illness and sickness – are reduced to the same root form) to preserve the accuracy of the textual data while preparing a database for running algorithms to generate both occurrence and co-occurrence matrices (for further details about this process, see Li et al., 2000; Recchia and Louwerse, 2015; Veronese et al., 2016, 2021).

The resulting qualitative database was composed of 1,616 occurrences, 652 raw forms, and 439 hapaxes (i.e., words that occurred once in the corpus). On adopting a threshold of at least four occurrences (text coverage 83%), root type/token ratio (an index of text richness) was 16.21, suggesting that the data were suitable for multiple correspondence analysis.

## Results

### Descriptive analysis and multiple correspondence analysis

The descriptive statistics for well-being scores by alexithymia group are summarized in Table 1.

**Table 1:** Main statistical descriptives for wellbeing scores in relation to TAS-20 cutoff scores (*N* = 229).

	Non-alexithymia (*n* = 107)		Not-defined (*n* = 37)		Possible alexithymia *n* = 85	
	*M*	*SD*	*M*	*SD*	*M*	*SD*
WHO-5 Scores	59.14	18.59	58.59	20.55	41.79	23.20

M = mean, SD = standard deviation. According to WHO, subjects scoring lower than 50 require screening for clinical depression.

**Figure 1:**
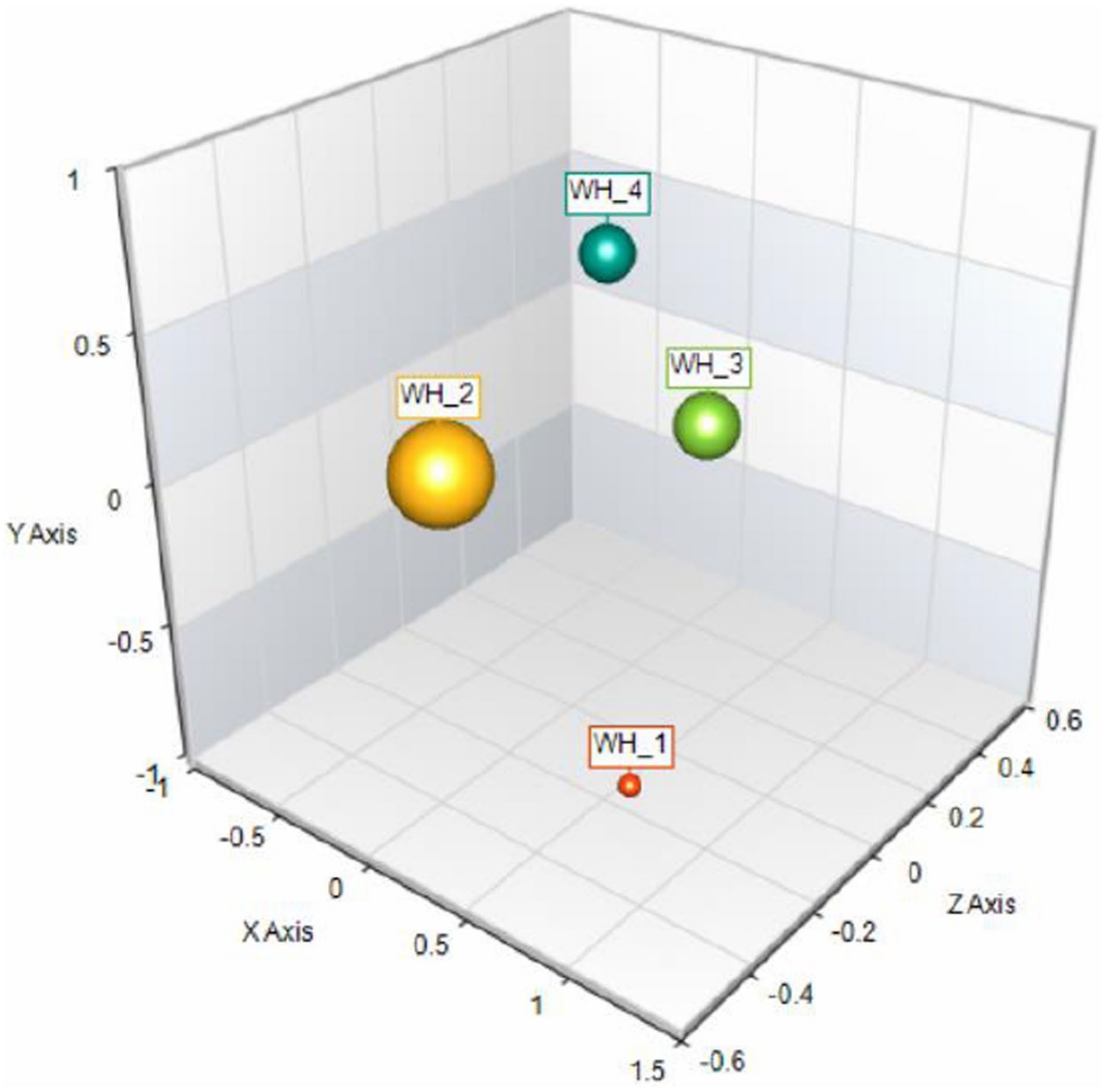
Graphical representation of percentage distribution of adolescents by WHO-5 scores: low wellbeing (red dot, < 25) 12.2%, medium-low wellbeing (yellow dot, WHO-5 between 26 and 50) = 33.6%, medium-high wellbeing (green dot, WHO-5 between 51 and 75) = 34.9% and high wellbeing (light blue dot, WHO-5 > 75) = 19.2%.

An analysis of variance in the wellbeing scores of the three different cohorts of participants grouped as a function of their levels of alexithymia revealed a statistically significant difference [*F* (2,227) = 18.45, *p* < 0.001]. Given that the three group sizes were quite different, we performed Levine’s test to assess the variances among groups. There was homogeneity of variances for scores, as supported by the test [Levene’s test =1.84, *p*=. 161]. Bonferroni post-hoc analyses revealed that the group of participants characterized by possible alexithymia reported statistically significant different scores (*p* < 0.001) than the other two groups. Group differences in terms of wellbeing scores suggested that the group with possible alexithymia reported lower scores when compared with both the non-alexithymia group (Δ = 17.35, 95% confidence interval = 10.09–24.61) and the not-defined group (Δ = 16.80, 95% confidence interval = 6.96–26.65). The effect size, Cohen’s d, for the three-group comparison was equal to .39. This outcome lent support to the idea that the group of adolescents with possible alexithymia symptoms experienced significantly lower wellbeing than the other two groups. See Figure 1 for the percentage of participants in each range of wellbeing scores.

**Figure 2:**
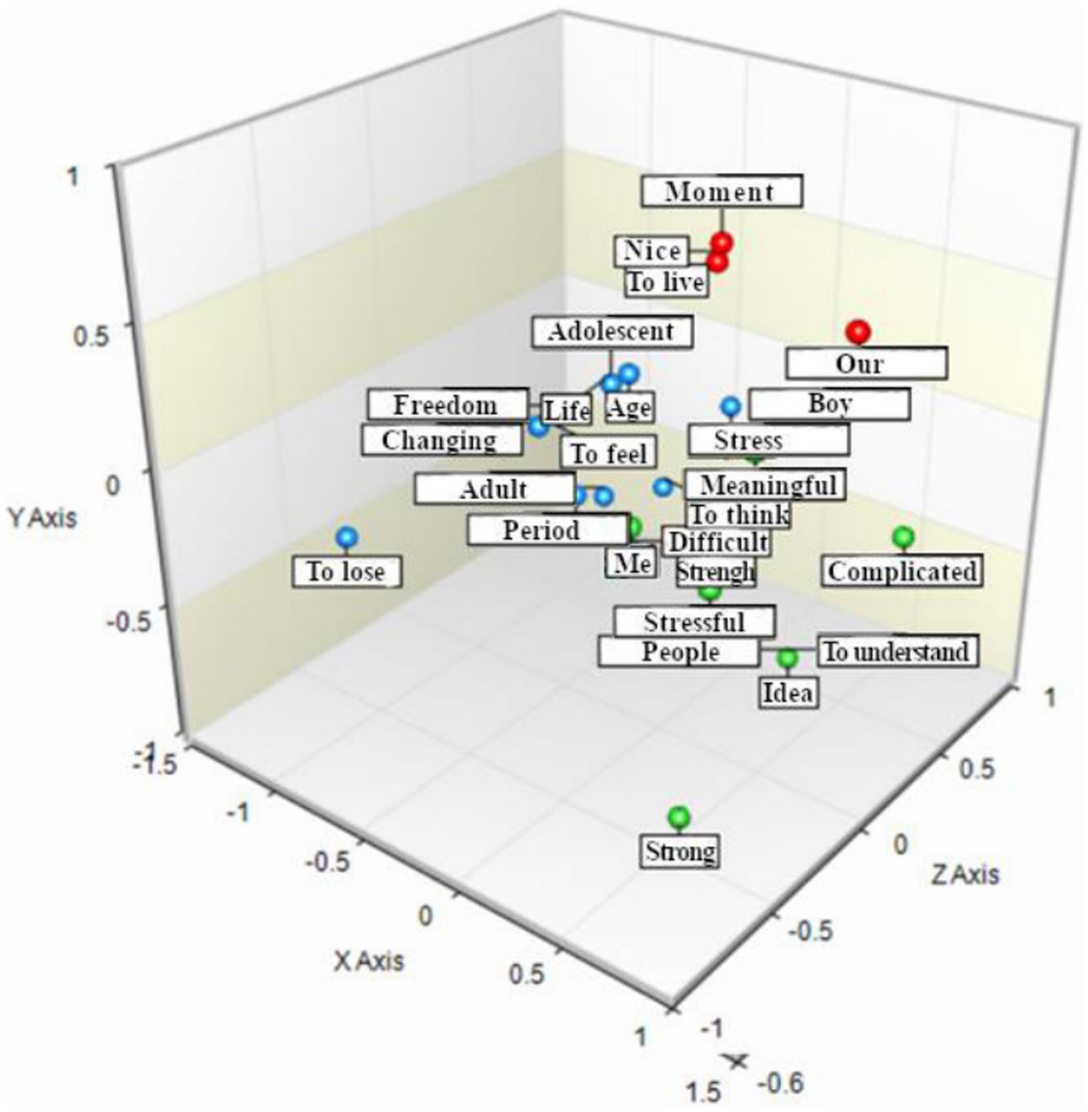
Graphical representation of the associations between words used to described adolescence and the well-being cohorts.

The next step in our data analysis strategy was to explore the association between scores on WHO-5 and occurrences of the words used by the participants to describe their adolescence via narrative metaphors. The results are summarized in Figure 2.

Starting from the group characterized by lower wellbeing (green dots), the most frequently used words were: strong (forte), stressed (stressato), complicated (complicato), difficult (difficile), to understand (capire), people (persone). The group reporting medium wellbeing (light blue dots) typically made use of words such as: change (cambiamento), age (età), to feel (sentire), to lose (perdere), adulthood (adulto). Finally, the words most frequently used by the group with a high level of wellbeing (red dots) were: good (bello), to live (vivere), moment (momento) and ours (nostro).

**Figure 3:**
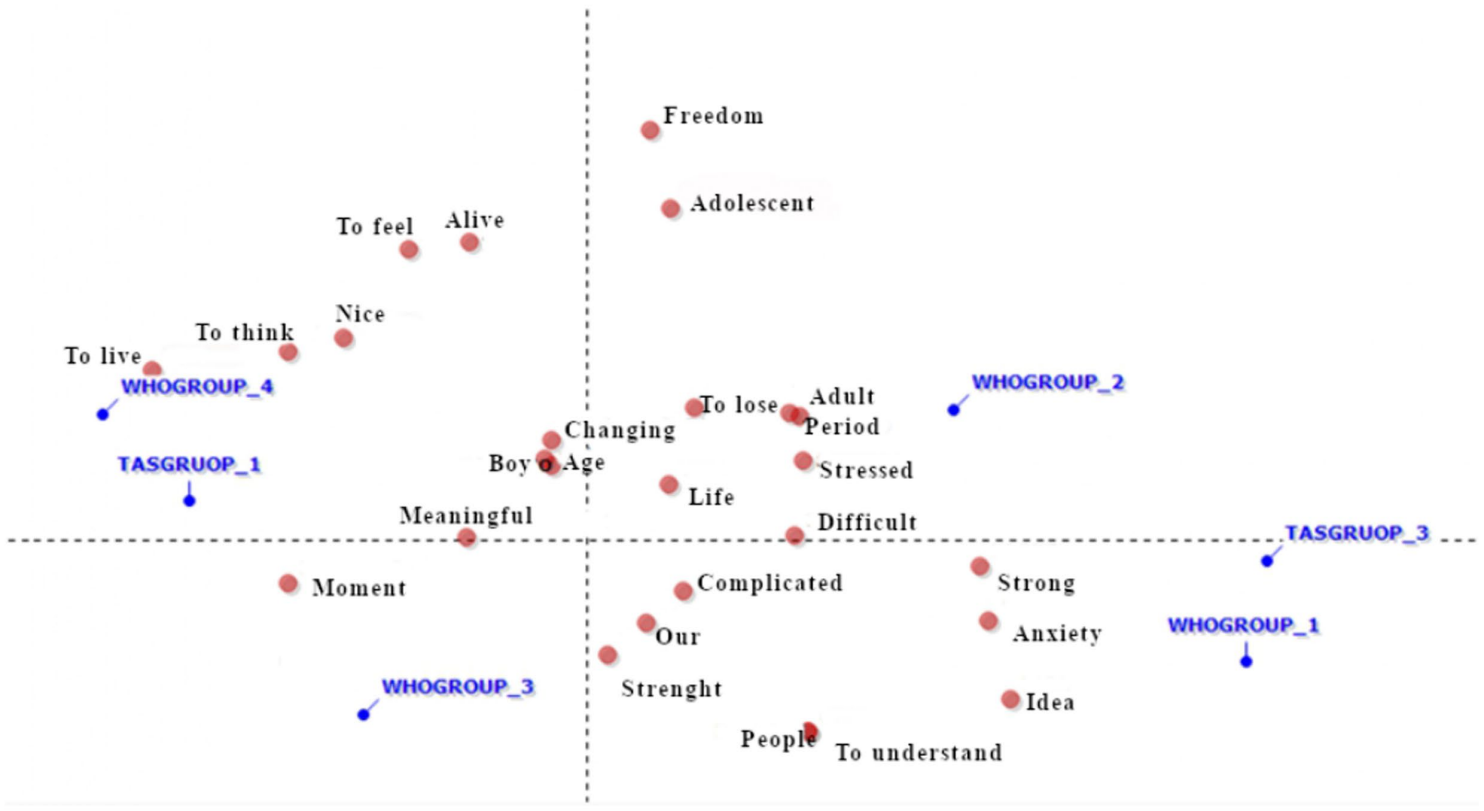
Results of multiple conespondence analysis.

Next, we conducted correspondence analyses with a view to producing an organized semantic space (see the Method section) that summarized the numerical relationships between the metaphors and the two selected explanatory variables: well-being and alexithymia. The results of the co-word analysis of correspondence are graphically represented in Figure 3.

Overall, the correspondence analyses suggested a two-dimensional (eigenvalue_dim1 = 0.68 and eigenvalue_dim2 = 0.53) solution accounting for approximately 48.9% of total inertia (that is to say, the conceptual equivalent of explained variance in multivariate analysis). As shown in Figure 3, the semantic space is represented two relatively dense semantic attractors (more specifically, in the dynamics of non-linear systems, an attractor is a dense point in space at which movement may or may not take place; like a magnet, it can draw objects into its sphere of influence; Guastello, 2010). Toward the left pole of the X axis, the first semantic attractor was characterized by words such as: good (*t* = 1.55), moment (*t* = 0.94), to live (*t* = 1.70), to think (*t* = 0.94), to feel (*t* = 0.95) and alive (*t* = 1.93). In addition, this pole was associated with high levels of wellbeing (WHOGROUP_4, t = 5.33, *p* < 0.01) and low alexithymia (TASGROUP_1, *t* = 8.78, *p* < 0.01). This semantic profile may be interpreted as describing participants characterized by both high levels of wellbeing and low alexithymia, who were experiencing good feelings and emotions about being an adolescent during the COVID-19 pandemic. With regard to the second attractor, the opposite side of the “X” axis grouped words such as: be strong (*t* = 1.39), anxiety (*t* = 1.27), complicated (*t* = 1.33), to understand (*t* = 0.72) and people (*t* = 0.70). Furthermore, this pole was associated with low wellbeing (WHOGROUP_1, *t* = 8.99, *p* < 0.01) and high alexithymia (TASGROUP_3, *t* = 11.38, *p* < 0.01). This semantic profile seemed to include words used by adolescents with low wellbeing and high alexithymia to express negative emotion and general difficulty understanding others (or being understood).

The final stage of the analysis involved attributing a meaning to the two Cartesian axes so as offer an overall description of the word profiles organizing the semantic space. This kind of analysis may be performed by first examining the geometric figures formed by the outermost points of the representation (see Greenacre, 2007): in the present case, a polygon whose vertices correspond to the positive and negative poles on the x-axis and y-axis. Next, the two dimensions were labeled based on the relative contributions of the different words to the various factors, as established by measuring the association between each of the root forms and its underlying semantic dimension. As in numerical principal component analyses, this was done by computing and comparing saturation coefficients – k –, which took the form of squared cosine values (i.e., a measure of association ranging between 0 and 1 that resembles a saturation coefficient, Benzecri, 1992). The first dimension, corresponding to the “X” axis, separated the high wellbeing/low alexithymia group on the left from the low wellbeing/high alexithymia group on the right side. The second dimension corresponded to the “Y” axis and featured an attractor describing negative personal feelings (negative y-axis) and, on the opposite side, positive internal states (positive y-axis). The words associated with the first dimension were: anxiety (cos = 0.52), stress (cos = 0.33), to live (cos = 0.41), strong (cos = 0.34) and difficult (cos = 0.23). It seems that here the latent component drew together different negative emotions and feelings connected with the experience of being an adolescent during the pandemic. From this perspective, the axes may be interpreted as reflecting an overall representation of internal states that ranges from positive psychological experience (negative side of x-axis) to negative outcomes (positive x-axis). The words associated with the second dimension were: alive (cos = 0.86), freedom (cos = 0.84), adolescent (cos = 0.75), to feel (cos = 0.66), to understand (cos = 0.65) and good (0.49). A closer look at the Cartesian coordinates reveals that this second dimension divides the metaphors of those who were more “agentic” (in other words who were able to articulate a sense of control over their lives) on the positive side of Y-axis, from the words of those who were more “passive” and felt that they needed to be strong in a complicated period. Finally, in keeping with the qualitative research tradition and in order to provide a flavor of the adolescents’ words, we report representative extracts from their narrative texts in Table 2. This is a double-entry table that represents the intersections between the high vs. low well-being groups (Groups 1 and 4) and high vs. low alexithymia groups (Groups 1 and 3).

**Table 2:** Selected extracts from the adolescents’ texts grouped by levels of wellbeing and alexithymia.

	WHO_GROUP1	WHO_GROUP4
TAS_GROUP1	No quotes available	“life is like a tightrope, be careful jumping because it might break.” (male, 14 y.o) “an explosion of beautiful and less beautiful emotions, and a desire to return to normal life” (Female, 16 y.o.) “be the future” (Male, 17 y.o.) “a colorful explosion” (female 15 y.o.)
TAS_GROUP3	“A storm of hormones, emotions and negative thoughts in the face of uncertainty and hope for a less bleak future” (male, 16 y.o.) “To be poised on a cliff called sociality.” (male, 15 y.o.) “Please help me” (female, 17 y.o.) “having the desire to experience, go out and enjoy life but at the same time the awareness that it is not always possible or not succeeding due to other causes (e.g., strict parents or lack of motivation)” (Male, 16 y.o.) “Our bodies are maturing with an emptiness inside, and that feeling will one day end with emotions inside.” (Female, 17 y.o.) “It would be too complicated to explain, amongst other reasons because at this time I do not understand anything about anything anymore” (Female, 15 y.o.)	“it is not a good period” (Male, 14 y.o.), “performance anxiety mixed with fun” (Male, 17 y.o.) “there’s a saying: yesterday is history, tomorrow is a mystery, but today is the present...that’s why it’s called the present.” (Female, 16 y.o.)

WHO_GROUP4 = high wellbeing, WHO_GROUP1 = low wellbeing, TAS_GROUP1 = no alexithymia, TAS_GROUP3 = possible alexithymia. The label “no quotes available” is used because the first quadrant comprised three missing answers and two “I do not know”.

## Discussion

The results of this study are generally in line with the hypotheses that we had formulated. They will be commented on below with reference to the two main objectives concerning the link between alexithymia and adolescents’ perceived well-being and the associations between these variables and their use of self-metaphors to represent themselves during the pandemic. We finally discuss on the use of narrative metaphors as an effective method of investigating well-being in adolescents along with more standardized instruments.

### Well-being and alexithymia

With regard to perceived levels of well-being among adolescents, we found that just over half of our sample reported medium-high or high levels, while 45.8% are below the cutoff score of 50 and 12.6% below the score of 25. Considering that, conventionally, scores between 30 and 50 correspond to mild depression and < 30 to moderate depression, these outcomes should make us reflect on the possible effects of the public health crisis on well-being in adolescence. This finding is in line with the existing literature, where the negative effects of the COVID-19 emergency on mental health have been extensively discussed. It is especially consistent with the results of a study in a large group of Austrian adolescents (Pieh et al., 2021), which points up, from 2018 to 2020, a decrease in the mean perceived well-being score, as measured by the WHO-5, from 43.7 to 35.8 (albeit that both of these scores invite reflection on the state of mental health in adolescence more generally). Data collected from samples of adolescents in previous years showed generally higher levels of perceived well-being (see, e.g., Strittmatter et al., 2015), suggesting that the public health crisis has likely accelerated a decline in adolescents’ mental well-being that has been showing up in the literature for the past 7 or 8 years (Inchley et al., 2020).

Both the group of adolescents with no alexithymic traits, as assessed using TAS-20, scores and the “not defined” group (with no clear alexithymic traits) enjoyed sufficiently good levels of perceived well-being. Perceived well-being in the group of adolescents with scores above the clinical cutoff for alexithymia was significantly lower than that of the other groups, with a mean score that fell within the range that would normally require screening for depression. This result is in line with evidence that highlights the link between alexithymia and depressive symptoms (Alkan Härtwig et al., 2013; Karukivi et al., 2010a,b), a relationship that appears to have become particularly pronounced during the COVID-19 emergency (Wang et al., 2021). Moreover, this result is in line with research that emphasizes the link between alexithymia and other clinical profiles, such as eating disorders and alexithymia (Meneguzzo et al., 2022), which the covid-19 experience has further intensified, as evidenced by the increase in restrictive and compensatory behaviors during the pandemic as a dysfunctional strategy to maintain or lose weight (Meneguzzo et al., 2024). Therefore, it seems that adolescents with marked difficulty identifying and being aware of their own emotions also need to invest significant effort in attempting to identify, understand, and manage sources of stress in complex situations, such as the present pandemic scenario. In line with the stress-alexithymia hypothesis (Martin and Pihl, 1985) and with studies associating alexithymic traits with the risk of burnout (Hamaideh, 2018; Farina et al., 2021), understood as a manifestation of fatigue in alexithymic young people, so too, our results clearly showed alexithymia to be associated with low subjective well-being and the risk of developing depressive symptoms.

### Metaphors of adolescence during the pandemic

The link between perceived well-being and alexithymia identified in this study is reinforced by additional insights given by the intersection of this association with recurrent themes in narrative metaphors on adolescence, as identified via co-word analysis.

A first interesting finding concerns the association between low perceived well-being and the use of words from the semantic domain of fatigue and difficulty (e.g., “stressed,” “complicated”...), while high levels of well-being are associated with terms that express qualities or positive and vital aspects of this period of life (e.g., “beautiful,” “live”...). If metaphors, as proposed by Lakoff and Johnson (1980), facilitate detection of the features of a concept that people identify as most salient, our data clearly implies that high levels of perceived well-being are associated with images of adolescence that emphasize vitality, despite teenagers’ peculiar life conditions at this historical moment. On the other hand, subjects whose self-perceived psychological well-being is poor, tend to emphasize the stress and difficulty associated with being adolescents. Superimposing the alexithymia cohorts on the semantic space built around the narrative metaphors suggests that subjects with alexithymic traits, together with those who display low levels of well-being, belong to a semantic area characterized by negative emotions and general passivity in coping with both these internal states and external stressors. It is plausible, again in alignment with the stress-alexithymia hypothesis, that poor emotional self-awareness and general difficulty in identifying feelings, contribute to an emphasis on fatigue, with respect to which a sense of being overwhelmed and unable to cope appears to emerge, as reflected in terms that articulate strategies of passive “resistance” to the situation (“strong,” “strength”) rather than an active quest for solutions. This, in turn, can understandably lead to a negative assessment of personal well-being. In contrast, the semantic space occupied by non-alexithymic adolescents with higher levels of well-being is characterized by terms that not only express positive emotions, but also greater agency (“freedom,” “understand”): it is possible that a greater ability to understand emotional experience facilitates a more complex reading of the contingent situation, and the identification of both possible sources of stress and vital resources. This view could in turn enhance subjects’ sense of self-efficacy in managing stressful situations, thereby allowing satisfactory levels of personal well-being to be maintained.

Relating representative extracts from the participants’ narrative metaphor texts to levels of well-being and alexithymia confirmed the above set of associations. More specifically, levels of well-being below the cutoff for screening for depression, when associated with alexithymia, seem to favor the emergence of descriptions in which confusion and the feeling of being emotionally overwhelmed prevail (“storm of hormones,” “please help me”). On the other hand, when poor wellbeing is not associated with alexithymia, depressive symptoms appear to dominate: indeed, the adolescents at the intersection of these two conditions did not even answer the question or wrote that they do not know how to answer it. On the contrary, when high levels of perceived well-being are associated with alexithymic traits, the texts, although focused on the theme of difficulty, do not seem to reflect a state of being emotionally overwhelmed or particular confusion, but rather use “catch phrases” to describe the adolescent condition. Finally, strong well-being in the absence of alexithymic traits, was associated with the use of evocative images representing vitality and a positive outlook on the future.

Overall, these novel findings appear to show an interactive effect of perceived well-being and alexithymia on adolescents’ ability to identify and describe their own condition. When their levels of well-being are low, adolescents seem to lack the motivation or the ability to identify an image that describes them, even if they do not display alexithymic traits: below a certain level of psychological well-being, consistently with a depression framework, the ability to identify and understand one’s own feelings appears to be less important, as though feeling bad were acting to block access to resources for emotional competence. On the other hand, when low levels of well-being are associated with alexithymic traits, the emergent image of adolescence is one of feeling confused and overwhelmed by emotions that are difficult to manage. In this case, it might be plausible to assume that, on the one hand, adolescents with clear basic alexithymic traits are conditioned by them in relation to their ability to read emotional experience, leading them to emphasize fatigue. On the other hand, when depressive symptoms are present, situations of emotional stress may accentuate alexithymic traits (see Wang et al., 2021). Hence, reciprocal interdependence among these aspects appears to be reflected in the ability to provide a description of self. When perceived well-being is high, alexithymic traits appear to impact more on the ability to express and communicate one’s feelings in a personalized way (as reflected in the use of catch phrases), than on the ability to identify positive and negative aspects of adolescence.

### Limitations and future directions

This study, similarly to others of its kind, features several limitations that should be noted. First, the study is cross-sectional, which means that the teenagers were questioned at a point in time when the COVID-19 epidemic was still ongoing. While this was congruent with the research aims, the research design offers no information about the dynamic evolution of the phenomena under observation, either in terms of well-being or in terms of the metaphors used to characterize adolescence. A second limitation concerns the fact that the interviews were administered online. Although this approach facilitated data collection at a time when mobility constraints and public health measures made it difficult to gather data directly in the field, it raises concerns regarding the sample’s effective representativeness. The most susceptible groups of teenagers or those with educational and economic difficulties may have had restricted access to the Internet and computer technologies, affecting their ability to respond the survey. This means that caution is required in generalizing our findings to all Italian teenagers. In the future, follow-studies within this line of inquiry should be conducted with larger samples and longitudinal designs in order to gain a clearer picture of the variables studied, in terms of both the stability of the identified associations and the scope for change, especially given the fact that adolescence is a period of transition and rapid transformation in general.

Future research endeavors can explore several avenues to deepen our understanding of the interplay between the explored variables, especially in the context of ongoing societal challenges after the COVID-19 epidemic. Firstly, longitudinal studies could be employed to track changes in alexithymic traits, well-being, and metaphorical expressions over time, providing insights into the dynamic nature of these associations during the transitional period of adolescence. Additionally, investigating the impact of intervention programs aimed at enhancing emotional awareness and expression in adolescents may offer valuable insights into potential avenues for improving well-being. Furthermore, there is a critical need to extend this research to clinical populations, such as adolescents diagnosed with anxiety disorders, eating disorders, or other mental health conditions. By embracing diverse research methodologies and considering the experiences of clinical populations, future studies can contribute to the development of effective interventions that promote positive mental health outcomes in adolescents facing specific mental health challenges.

## Data availability statement

The raw data supporting the conclusions of this article will be made available by the authors, without undue reservation.

## Ethics statement

The studies involving humans were approved by Ethical Committe of Milano-Bicocca University. The studies were conducted in accordance with the local legislation and institutional requirements. Written informed consent for participation in this study was provided by the participants’ legal guardians/next of kin.

## Author contributions

EF: Conceptualization, Data curation, Formal analysis, Funding acquisition, Investigation, Methodology, Project administration, Resources, Software, Supervision, Validation, Visualization, Writing – original draft, Writing – review & editing. AP: Conceptualization, Data curation, Formal analysis, Funding acquisition, Investigation, Methodology, Project administration, Resources, Software, Supervision, Validation, Visualization, Writing – original draft, Writing – review & editing.
